# Diversity of Volatile Aroma Compound Composition Produced by Non-*Saccharomyces* Yeasts in the Early Phase of Grape Must Fermentation

**DOI:** 10.3390/foods11193088

**Published:** 2022-10-05

**Authors:** Doris Delač Salopek, Ivana Horvat, Ana Hranilović, Tomislav Plavša, Sanja Radeka, Igor Pasković, Igor Lukić

**Affiliations:** 1 Institute of Agriculture and Tourism, Karla Huguesa 8, 52440 Poreč, Croatia; 2 Department of Wine Science, The University of Adelaide, Urrbrae, SA 5064, Australia; 3 Centre of Excellence for Biodiversity and Molecular Plant Breeding, Svetošimunska 25, 10000 Zagreb, Croatia

**Keywords:** non-*Saccharomyces* yeasts, sequential inoculation, volatile aroma compounds, esters, GC/MS, Malvazija istarska wine

## Abstract

There is a lack of studies evaluating the metabolic contribution of non-*Saccharomyces* yeasts in early fermentation phases. This study aimed to investigate the volatile aroma profiles produced by various non-*Saccharomyces* yeasts just before sequential inoculation with *Saccharomyces cerevisiae* to provide an insight into the particular effects they induce at this stage. The grape must of Malvazija istarska was inoculated with monocultures of *Torulaspora delbrueckii*, *Metschnikowia pulcherrima*, *Pichia kluyveri*, *Lachancea thermotolerans*, and *Schizosaccharomyces pombe*, alongside a *S. cerevisiae* control. Eighty volatile compounds were quantified via headspace solid-phase microextraction and gas chromatography–mass spectrometry, and the data were statistically elaborated. Volatile profiles of non-*Saccharomyces* yeasts differed significantly from the *S. cerevisiae* control. Most treatments caused increases in linalool and β-damascenone, decreases in higher alcohols and fatty acids, and improved synthesis of odoriferous esters. *Torulaspora delbrueckii* and *M. pulcherrima* produced compounds not commonly found in *S. cerevisiae* fermented wines. Multivariate statistical analysis linked the investigated yeasts to specific, particularly abundant compounds. Future studies should explore to what degree these contributions persist after sequential inoculation with *S. cerevisiae* in diverse grape must matrices.

## 1. Introduction

Winemaking dates back to the beginning of civilization, but the scientific community keeps pursuing new technologies to improve the production and quality of wine. Although the yeasts that are usually selected for winemaking are from the genus *Saccharomyces*, most often of *Saccharomyces cerevisiae* species, there is great potential in the use of non-*Saccharomyces* yeasts. Non-*Saccharomyces* yeasts play an important role in the pre-fermentative stage of winemaking (e.g., cold soak) and un-inoculated fermentations, characterized by the coexistence and succession of multiple yeast species and strains [1]. Recently, several non-*Saccharomyces* yeasts with great oenological potential are commercially available, and this is likely to continue in the near future [2].

As most non-*Saccharomyces* yeasts show limited fermentation aptitudes, sequential or co-inoculation with *Saccharomyces* yeasts is required for fermentation completion. The sequential fermentation is initiated by a high concentration of a non-*Saccharomyces* species. For example, after a certain period of time or when the ethanol level reaches the desired level, *Saccharomyces* yeast is inoculated. In this manner, enough time is provided for the metabolic contribution of non-*Saccharomyces* yeasts before being inhibited by *Saccharomyces* yeasts and increasing alcohol levels, whilst avoiding stuck or sluggish fermentation [3].

Fermentation involving non-*Saccharomyces* yeasts may bring many advantages to the wine production process and the final wine quality, depending on the yeast genus, species and strain. It is a potential solution to reduce ethanol content in wine when mitigating the effects of climate change and the premature ripening of grapes, as well as producing wines with less alcohol to meet specific consumer and stylistic requirements. 

It has been shown that *Metschnikowia pulcherrima* [2] and *Lachancea thermotolerans* [4], in combination with *S. cerevisiae*, are good candidates for decreasing wine ethanol content. *Schizosaccharomyces pombe* is the highest malic acid consumer among yeasts [5] and thus can be used to decrease wine acidity through maloalcoholic fermentation [6]. On the other hand, *L. thermotolerans* shows the potential to increase total wine acidity through the production of lactic acid [4]. In excess, acetic acid has a negative impact on fermentation and wine aroma. Although *S. pombe* shows a tendency to produce high levels of acetic acid, an interesting strategy with sequential fermentation of *L. thermotolerans* and *S. pombe,* proposed as an alternative to malolactic fermentation, resulted in decreased acetic acid content [7]. High sulphite levels added in various stages of winemaking may cause consumer rejection. Application of non-*Saccharomyces* yeasts, in particular *Torulaspora delbrueckii* and *M. pulcherrima*, in bioprotection by colonizing the environment in pre-fermentative stages and thereby limiting the development of the potentially undesirable microbiota, offers an alternative to SO_2_ [8]. Moreover, non-*Saccharomyces* yeasts can positively affect wine protein stability, either by producing more mannose-containing polysaccharides, which act as stabilizers or by acting proteolytically and reducing the levels of pathogenesis-related proteins [9].

One of the most important applications of non-*Saccharomyces* yeast is their use for obtaining wines with distinct aroma profiles. Non-*Saccharomyces* yeasts are a great source of different exogenous enzymes that may affect the release of various grape-derived compounds during fermentation, including terpenes, norisoprenoids, and thiols that have a positive impact on wine aroma. Their influence is more direct through the production of odoriferous fermentation aroma compounds, such as higher alcohols, fatty acids, and esters, in quantities and ratios which often differ among the species and from those obtained by *S. cerevisiae* [7]. 

Non-*Saccharomyces* yeasts affect the wine’s volatile composition in a species/strain-specific manner. For example, *P. kluyveri* and *M. pulcherrima* [10] decreased, while *T. delbrueckii* [11,12,13] and *L. thermotolerans* [13] increased the concentration of 2-phenylethanol in sequential or co-inoculation compared to pure *S. cerevisiae* fermentation. Although non-*Saccharomyces* species may have a positive impact on wine profile, there are also studies reporting the opposite, so the results were in many cases contrasting, even for the same yeast species. *Pichia kluyveri* was shown to be able to both reduce [10,14] and increase [15] the concentration of hexanol in co-fermentation with *S. cerevisiae*. Similar was observed for *T. delbrueckii,* causing either a decrease [11,12] or an increase [13,16] in isoamyl acetate concentration. In mixed cultures, non-*Saccharomyces* yeasts can modulate wine aroma by their own activity but also by changing the genomic expression of *S. cerevisiae* while coexisting during wine fermentation [17]. Multiple factors, such as the genetic predispositions of a particular non-*Saccharomyces* yeast, the availability of yeast nutrients, and the general composition of grape must certainly play a significant role in determining the outcomes of such fermentations. Considering such interactions and the fact that most previous studies investigated the influence of co-fermentation of non-*Saccharomyces* and *S. cerevisiae* yeasts based on the aroma of the final wine, the actual aromatic potential of non-*Saccharomyces* yeasts in co-fermentations was often not distinguishable. 

On the other hand, studies that investigated volatile profiles of pure culture fermentations of various non-*Saccharomyces* yeasts were mainly conducted at the end of monoculture fermentations [4,18,19,20]. However, this is not representative of their oenological application, as they are exclusively used in mixed cultures with *S. cerevisiae.* Indeed, the production of volatile compounds during fermentation fluctuates [21], and the final volatile profiles of non-*Saccharomyces* monocultures do not necessarily reflect their status at earlier fermentation stages (e.g., when *S. cerevisiae* is added). To our knowledge, only one study compared early fermentation volatile profiles of inoculated non-*Saccharomyces* yeasts, highlighting the unique behaviour of each yeast [22], which warrants further research. 

The main premise of this study was that in the initial phase of alcoholic fermentation, the species-specific effects of non-*Saccharomyces* yeasts on the grape must’s volatile profile would be more distinguishable than in the finished wines produced by mixed fermentation with *S. cerevisiae*. Therefore, the aim was to investigate the production of the volatile aroma compounds by five commercially available non-*Saccharomyces* yeasts in the early stage of fermentation, before the inoculation of *S. cerevisiae*. In this manner, their effect would be more distinguishable compared to finished wines produced by mixed fermentations with *S. cerevisiae* and their monoculture fermentations. 

## 2. Materials and Methods

### 2.1. Vinification

For this experiment, the grapes of Malvazija istarska (*Vitis vinifera* L.), the most spread and important native white grape cultivar in Croatia, were handpicked from the experimental vineyard of the Institute for Agriculture and Tourism in Poreč situated in the region of Istria, Croatia. Before grape processing, the equipment was cleaned with caustic soda solution and washed off, and then sanitized with an aqueous solution of potassium metabisulfite and citric acid and washed off again. The tanks were additionally washed with 70% ethanol. All the equipment was carefully and thoroughly washed off with hot water before use. The grapes were destemmed, crushed, and pressed immediately after harvest using a closed-type pneumatic press of 500 L capacity with the pressures of 2 × 0.5 bar and 1 × 0.8 bar (Letina Inox d.o.o., Čakovec, Croatia). The obtained juice was sulfited and cold-settled with the aid of Endozym Rapid pectolytic enzymes at 2 g/hL (AEB s.p.a. Brescia, Italy) for 48 h at 10 °C. The grape must had total acidity of 4.7 g/L, pH of 3.41 and 22.1 Brix˚. The total acidity was adjusted by adding 1.3 g/L of tartaric acid to obtain the concentration of 6 g/L; after the addition, the pH was set to 3.27. The must was distributed in 80 L stainless steel tanks and inoculated with yeast to start the fermentation. All fermentations were performed at 17 °C in triplicates. Diammonium phosphate (Corimpex Service Srl, Romans d’Isonzo, Italy) was added at 30 g/hL 36 h after inoculation. The concentration of sugars was monitored daily by a portable density meter DMA 35 (Anton Paar, Graz, Austria). After measuring the sugar concentration, the alcohol content was estimated based on the conversion table by Ribéreau-Gayon et al. [23]. When the alcohol level reached approximately 1.5–2%, fermented samples were collected for analysis.

### 2.2. Preparation of the Yeasts

Five non-*Saccharomyces* yeasts were used in this experiment: *T. delbrueckii* (BIODIVA^®^), *M. pulcherrima* (FLAVIA^®^), and *L. thermotolerans* (LAKTIA^®^) were purchased from Lallemand Inc. (Montreal, Canada), *P. kluyveri* (Frootzen^®^) was purchased from CHR Hansen (Hoersholm, Denmark) and *S. pombe* (Atecrem 12H^®^) was purchased from BioEnologia 2.0 (Oderzo, Italy). *Saccharomyces cerevisiae* var. *bayanus* (Lalvin EC1118^®^) purchased from Lallemand Inc. was used as a control.

*Torulaspora delbrueckii*, *M. pulcherrima*, *L. thermotolerans*, and *S. cerevisiae* were rehydrated according to the manufacturers’ protocols, while *S. pombe* in cream form and *P. kluyveri* frozen at −45 °C were added directly to the must. *Torulaspora delbrueckii*, *M. pulcherrima*, *L. thermotolerans*, *S. pombe*, and *S. cerevisiae* yeasts were added in the amounts recommended by the producers, which corresponded to the cell density of approximately 4–5 × 10^6^ cells/mL. The cell density of *P. kluyveri* recommended by the producer is much lower, 1 × 10^5^ cells/mL, but in this work, approximately 1 × 10^6^ cells/mL were inoculated to keep a similar order of magnitude as for other yeasts. 

### 2.3. Analysis of Volatile Aroma Compounds by Headspace Solid-Phase Microextraction and Gas Chromatography-Mass Spectrometry

Volatile aroma compounds were extracted from grape must by headspace solid-phase microextraction (HS-SPME) by the modified method proposed by Bubola et al. [24]. Prior to analysis, 7 µL of 5% sulfurous acid (Agrolit, Litija, Slovenia) and 50 µL of sodium azide (VWR BDH Prolabo, Radnor, SAD) were added to inhibit oxidation and microbial activity, respectively, and samples were centrifuged at 4000 rpm at 4 °C for 5 min using a laboratory centrifuge Universal 320 R (Hettich, Westphalia, Germany). Half a milliliter of the supernatant was placed in a 10 mL glass vial containing 3.45 mL of deionized water. A gram of ammonium sulphate and 50 μL of internal standards solution (2-octanol at 0.84 mg/L, 1-nonanol at 0.82 mg/L, and heptanoic acid at 2.57 mg/L) were added. The samples were incubated for 15 min under stirring at 800 rpm, and the extraction using a divinylbenzene/Carboxen/polydimethylsiloxane (DVB/CAR/PDMS; StableFlex, 50/30 μm, 1 cm; Supelco, Bellafonte, PA, USA) fiber took place for 40 min at 40 °C. When the extraction finished, the fiber was inserted into the GC/MS injector port at 248 °C for 10 min, with the first 3 min in splitless mode. 

For the identification and quantification of volatile aroma compounds a Varian 3900 gas chromatograph (GC) coupled to a Varian Saturn 2100T ion trap mass spectrometer (MS) (Varian Inc., Harbour City, CA, USA) was used. The GC-MS was equipped with an Rtx-WAX capillary column of the following dimensions: 60 m × 0.25 mm i.d. × 0.25 μm d.f. (Restek, Belafonte, PA, USA). The initial column temperature was 40 °C, then it was increased at 2 °C/min to 240 °C, and it remained at this temperature for the next 10 min. The carrier gas was helium with a 1.2 mL/min flow rate. Electron ionization mode (EI, 70 eV) in the range of 20–350 *m/z* was used to acquire mass spectra. 

A comparison of retention times and mass spectra with those of the pure standards and with those available in the NIST05 library was used for identification. Spectra reverse match numbers RM > 800 were considered satisfactory. In the cases of RM < 800, the identification was based on the similarity of the intensities of a quantifier ion and other major ions in the spectra to those in the reference spectra. A solution containing C10 to C28 n-alkanes was injected under the same chromatographic conditions, the linear retention indices were calculated, and the identity of volatile compounds was additionally confirmed by comparison with the retention indices reported in the literature. Standard solutions were also injected, and the calibration curves were constructed with *r*^2^ > 0.99 in all cases. Internal standards were used for normalization before quantification by using calibration curves. The compounds present in high concentrations were quantified based on total ion current peak area, while quantifier ions were used to quantify others. Method validation results were previously published in the study of Bubola et al. [24]. Compounds for which the authentic standards were not available were semi-quantified as equivalents of the corresponding internal standards. A response factor equal to one was used.

### 2.4. Statistical Data Elaboration

One-way analysis of variance (ANOVA) and the Least Significant Difference (LSD) test (*p* < 0.05) were used to determine statistically significant differences between the treatments. After normalization, forward stepwise linear discriminant analysis (SLDA) and hierarchical clustering analysis (HCA) were applied to 40 volatile compounds with the highest Fisher ratio values (*F*-ratios) obtained by ANOVA. Wilk’s lambda was used as a selection criterion in SLDA, with *F*-value to enter = 1 and *F*-value to remove = 0.5. Statistica v. 13.2 software (StatSoft Inc., Tulsa, OK, USA) was used for ANOVA and SLDA. MetaboAnalyst v. 5.0 [25] was used for generating box plots and performing HCA using the Ward algorithm and Euclidean distance analysis.

## 3. Results and Discussion

### 3.1. Fermentation Dynamics

**Figure 1:**
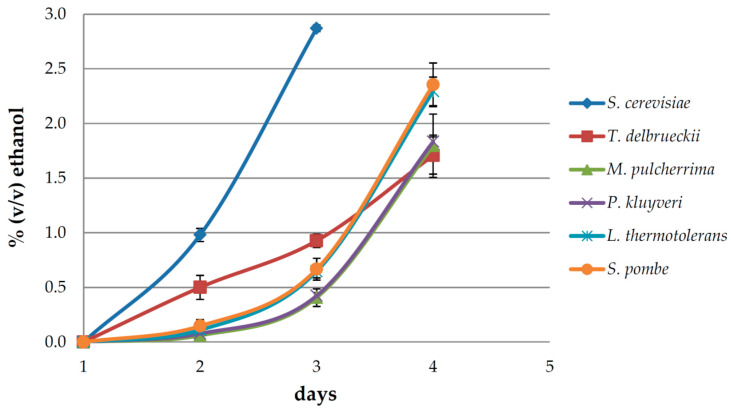
Ethanol production over time in early fermentation of Malvazija istarska (*Vitis vinifera* L.) white grape must inoculated with *Saccharomyces cerevisiae* and five non-*Saccharomyces* yeasts: *Torulaspora delbrueckii*, *Metschnikowia pulcherrima*, *Pichia kluyveri*, *Lachancea thermotolerans*, and *Schizosaccharomyces pombe*.

The dynamics of the production of ethanol in the early fermentation phase after inoculation with the six investigated yeast species are shown in Figure 1. *Sacharomyces cerevisiae* was the first to reach and even exceed the target ethanol level in three days. *Metschnikowia pulcherrima*, *P. kluyveri*, *L. thermotolerans*, and *S. pombe* exhibited similar patterns with a relatively slow start during the first three days, followed by accelerated fermentation/ethanol production on day four, when they were sampled. Interestingly, *T. delbrueckii* followed a different course, starting more intensively than the other non-*Saccharomyces* yeasts, but keeping approximately the same pace until the fourth day. Since it was practically impossible to sample the ferments at exactly the same point of fermentation, the ethanol levels produced slightly differed among the investigated yeasts, which possibly had a small impact on the concentrations of volatile aroma compounds released and produced in this phase.

### 3.2. Volatile Aroma Compounds

The concentrations of volatile compounds produced in the early phase of fermentation Malvazija istarska grape must by various non-*Saccharomyces* yeasts, and a *S. cerevisiae* monoculture control are reported in Table 1. A total of 80 compounds were identified, including 11 terpenes, five C_13_-norisoprenoids, nine alcohols, six acids, 40 esters, and nine miscellaneous compounds. Statistically significant differences between various yeasts were found for the majority of compounds.

#### 3.2.1. Terpenes

Terpenes originate from grapes and are considered among the leading carriers of varietal aroma, especially in wines containing higher concentrations, where they play a significant role in determining varietal typicity. Monoterpenols, such as geraniol, citronellol, nerol, ho-trienol, and especially linalool, are the most relevant terpenes in wine in sensory terms, imparting positive floral and fruity notes [26]. Several previous reports emphasized a significant contribution of linalool to Malvazija istarska varietal aroma, with concentrations repeatedly higher than its odor detection threshold [27]. Although it is generally considered that terpenes are less influenced by fermentation parameters, the enzymatic activity of yeast can influence their behaviour during fermentation and their composition in the final wine, principally by affecting the release of their free volatile forms from the corresponding glycosides [28]. Moreover, terpenes undergo numerous interconversions during fermentation, which can also be affected by the yeast species [29]. 

Linalool was the only major monoterpenol identified in this study. The highest concentration was found in the must inoculated with *P. kluyveri* yeast (Table 1, Figure S1). Several studies reported limited or no effects of *P. kluyveri* in sequential fermentation on the content of total terpenes when compared to pure *S. cerevisiae* inoculation [10,15], presumably because of its limited β-glucosidase activity [30]. However, the concentrations of particular monoterpenes, such as ho-trienol, were found to be positively affected [10].

The lowest concentration of linalool, although not significantly different from that found in some other treatments, was found in the control *S. cerevisiae* must, which was in line with the relatively low β-glucosidase activity of this commercial *S. cerevisiae* (ex-*bayanus*) strain, as shown earlier [31]. Other yeast species investigated in this study were previously shown to increase the content of certain terpenes in sequential or co-inoculations compared to *S. cerevisiae* monoculture. 

Azzolini et al. [11] recorded an increase in α-terpineol¸ ho-diendiol I, and endiol concentrations after sequential fermentation with *T. delbrueckii* in red wine, while Čuš and Jenko [32] observed an increase in linalool and a decrease in citronellol and geraniol concentration after a similar experiment with the same yeast. Linalool concentration increased after *T. delbrueckii* monoculture inoculation in the early phase of fermentation (2–3% *v/v* ethanol), as reported by Beckner Whitener et al. [22]. In this work, early fermentation *T. delbrueckii* must contained the lowest concentration of geranyl acetate among all the treatments, as well as a lower concentration of β-pinene and a higher concentration of eucalyptol in relation to *S. cerevisiae* must (Table 1). *Torulaspora delbrueckii* was previously highlighted as yeast capable of inducing interconversion reactions between monoterpenes [29], so it is possible that this phenomenon also had an effect in this study.

In previous studies, *L. thermotolerans* inoculation exhibited a positive effect on the concentrations of nerol and 4-terpineol in monoculture [22], as well as of geraniol, farnesol, and citronellyl acetate in sequential fermentation with *S. cerevisiae* [33], implying a significant β-glucosidase activity. In this study, *L. thermotolerans* did not have an effect on terpene concentrations when compared to *S. cerevisiae* control. Similar was observed for *M. pulcherrima* (Table 1), contrary to a previous investigation where it positively affected the content of linalool in early fermentation [22].

The highest concentrations of several other monoterpenes, such as camphene, β-pinene, menthol, 6,10-dihydromyrcenol, and geranyl acetate were found in *S. pombe* inoculated must (Table 1, Figure S1).

#### 3.2.2. C_13_-Norisoprenoids

Like terpenes, C_13_-norisoprenoids are not products of fermentation. They are formed by degradation of carotenoid precursors whose amounts are mainly predetermined by pedoclimatic and grape growing conditions in a vineyard, as well as pre-fermentation grape processing steps and parameters, such as harvest, transport, crushing, pressing, etc. [34]. However, the degree of liberation of free volatile C_13_-norisoprenoids during fermentation can also be conditioned by yeast [35]. The most important C_13_-norisoprenoids in grapes and wine are β-damascenone, due to its extremely low odor detection threshold [26], and, to a lesser degree, β-ionone. Both are considered positive contributors due to pleasant odors they produce, which are reminiscent of stewed apple and violet flowers, respectively.

The highest concentration of β-damascenone was found in *P. kluyveri* must, although not significantly different than that found in *L. thermotolerans* and *S. pombe* inoculated musts (Table 1, Figure S1). *Torulaspora delbrueckii* and *M. pulcherrima* early must ferments also contained concentrations higher than that found in *S. cerevisiae* control, which was in line with previous findings [22]. The lowest concentration of β-damascenone in *S. cerevisiae* must corroborated the relatively low enzymatic activity of the yeast strain used [31]. *S. pombe* must contained higher concentrations of other C_13_-norisoprenoids compared to *T. delbrueckii* must, with all of them being β-ionone derivatives (Table 1). 

#### 3.2.3. C_6_-Alcohols

C_6_-alcohols are formed by degradation of lipids, i.e., long-chain fatty acids, in a series of enzymatic reactions during harvest and pre-fermentation grape-processing steps. A fraction is present in grapes and transfers to grape must and wine in both free or glycosidically bound form, where the latter can be cleaved by the action of (yeast) enzymes to release free volatile molecules. Another small portion of a major C_6_-alcohol 1-hexanol is formed in fermentation. Although these compounds are often mentioned among the possible negative contributors to wine aroma by their herbaceous odors, they rarely have an impact due to relatively high odor perception thresholds [26]. 

Since, in this study, the same homogenized must was used, the differences in C_6_-alcohol concentrations were a result of differential yeast activity. The differences observed were marginal but statistically significant for all the identified C_6_-alcohols. Hexanol was found in higher concentration in *T. delbrueckii*, *M. pulcherrima*, and *L. thermotolerans* than in *S. pombe* and control *S. cerevisiae* musts. Similar was observed for *cis*-3-hexen-1-ol, while *T. delbrueckii* must was the most abundant in *trans*-3-hexen-1-ol. The highest concentration of *cis*-2-hexen-1-ol was found in *S. pombe*. In previous studies, *P. kluyveri* was shown to be able to both reduce [10,14] and increase the concentration of hexanol in sequential fermentation compared to *S. cerevisiae* alone [15].

#### 3.2.4. Higher Alcohols

Wine major higher alcohols are formed in fermentation by yeast either from sugars or from amino acids by the Ehrlich mechanism [17]. They form a basis of wine aroma and, at a low concentration, contribute to its complexity and character, while at total levels above 350 mg/L can have a direct negative impact and also mask other, positive aromas. The importance of higher alcohols is not solely in their contribution to wine aroma as they are also precursors to particular odoriferous volatile esters.

The highest concentration of the major wine higher alcohol, i.e., isoamyl alcohol was detected in the control *S. cerevisiae* must, although not significantly different compared to the levels found in *P. kluyveri* and *L. thermotolerans* inoculated musts (Table 1, Figure S2). It was shown in previous studies that particular non-*Saccharomyces* yeast, such as *P. kluyveri* [10] and *M. pulcherrima* [10,36], produce lower concentrations of higher alcohols in sequential fermentation compared to pure *S. cerevisiae* inoculation, although opposite results were also published for co-inoculation with *M. pulcherrima* in red wine [37]. In this work, a lower concentration of isobutanol was recorded in *M. pulcherrima* and the lowest in *T. delbrueckii* must in relation to the other musts (Table 1). It was shown previously that the application of different commercial preparations, and therefore different strains of the same non-*Saccharomyces* yeast, e.g., *T. delbrueckii,* in sequential fermentation can lead to contrasting outcomes regarding the content and composition of higher alcohols in relation to pure *S. cerevisiae* inoculation [11,38]. Besides the strain, the availability of yeast nutrients and suppressors in a given grape must matrix, as well as the genetically predetermined regulation system for the selection of nitrogen from various sources, certainly have a large effect [18].

In contrast to other major higher alcohols in wine, 2-phenylethanol is a carrier of a pleasant odor reminiscent of roses. It is mainly a product of alcoholic fermentation, although a smaller part derives from grapes in both free and glycosidically bound form. The concentration found in the control must inoculated with *S. cerevisiae* almost doubled those found in the musts of non-*Saccharomyces* starters (Table 1, Figure S2). As in the case of the abovementioned other major fermentation alcohols, previous results regarding the effect of non-*Saccharomyces* yeasts in sequential or co-inoculation with *S. cerevisiae* on 2-phenylethanol concentration were also contrasting. While certain authors observed a decrease after the use of *P. kluyveri* and *M. pulcherrima* [10], others noted an increase after inoculation with *T. delbrueckii* [11,12,13] and *L. thermotolerans* [13]. 

#### 3.2.5. Volatile Acids

Volatile acids are known to impart mostly undesirable odors, described as vinegary in the case of acetic acid and fatty, cheesy, and rancid in the case of short- and middle-chain volatile fatty acids. The level of the major volatile acid in wine, acetic acid, was higher in *P. kluyveri* than in *T. delbrueckii* inoculated must. *S. pombe* is generally known for generating high levels of acetic acid [39], which was not the case at the early fermentation stage in this study (Table 1). On the other hand, one of the advantages of using *T. delbrueckii* as a co-starter is the reduction of volatile acidity compared to standard *S. cerevisiae* monoculture fermentations [12,40]. The results of this investigation partially corresponded to this hypothesis, since the concentrations of the majority of volatile acids, including acetic acid (although without a statistically significant difference), as well as total fatty acids, were lower in *T. delbrueckii* than in *S. cerevisiae* inoculated must.

*Saccharomyces cerevisiae* produced the highest and *T. delbrueckii* the lowest concentrations of the majority of middle-chain fatty acids. The exception was hexanoic acid, which was the highest in *S. pombe* must (Table 1, Figure S2). *Pichia kluyveri* was previously shown not to affect [10] or even reduce the concentrations of volatile fatty acids, especially decanoic acid [15]. 

#### 3.2.6. Esters

Esters are among the most important contributors to wine aroma with their positive fruity and floral odors. They are mainly formed during alcoholic fermentation by yeast but can also be synthesized by bacteria during malolactic fermentation and via chemically induced esterification during wine aging. Several yeast enzymes are involved in the biosynthesis of esters, and their activity is mainly determined by the expression of corresponding genes [18]. The variability in ester-related enzymatic activities among *Saccharomyces* yeasts was already described [41] and suggested for non-*Saccharomyces* yeasts. The final concentration of esters in the wine after alcoholic fermentation is determined by the competing activity of ester-synthesizing enzymes and esterases from yeasts that can synthesize esters but are mostly responsible for their cleavage [42]. It is known that extracellular esterases are present in *S. cerevisiae* [43], while non-*Saccharomyces* yeasts still need to be investigated more from this aspect [30]. Another factor that strongly influences the content and composition of esters in wine is the composition of the grape juice matrix, especially the availability of substrates generated in carbon, nitrogen, and fatty acid metabolism [18].

Two main classes of esters are formed in wine: the ethyl esters, which are esters of ethanol and fatty acids, and the acetate esters, which are esters of higher alcohols and acetic acid. The concentration of the main wine ester, ethyl acetate, was higher in *S. cerevisiae* and *T. delbrueckii* than in *P. kluyveri*, *S. pombe*, and especially *M. pulcherrima* must, which contained the lowest concentration (Table 1, Figure S3).

The concentration of ethyl propanoate differentiated well the early fermentation profiles of the investigated yeasts. The highest concentration was found in *T. delbrueckii*, followed by *S. cerevisiae*, *L. thermotolerans*, and *P. kluyveri*, with the lowest concentrations found in *M. pulcherrima* and *S. pombe* musts which did not differ among each other (Table 1, Figure S3). *Torulaspora delbrueckii* was also relatively abundant in ethyl isobutyrate when compared to the other musts. Such results are in accordance with previous studies that observed an increase in these esters in mixed *T. delbrueckii/S. cerevisiae* fermentations [13,16]. Odd-chain and branched-chain fatty acids, which served as precursors to the mentioned ethyl esters, are not formed from acetyl-CoA through the fatty acid synthase (FAS) complex but from the degradation of threonine and valine, respectively, via the Erlich pathway [21], so the results obtained implied particular differences in amino acid metabolism between the investigated yeast. 

Linear even-chain fatty acid ethyl esters are some of the most important positive contributors to wine aroma due to their low perception thresholds and relatively high concentrations. The highest concentration of ethyl butyrate was found in *S. pombe*, *S. cerevisiae*, and *L. thermotolerans* musts, while *T. delbrueckii* must was the least abundant in this, as well as in middle-chain ethyl esters formed through the FAS complex, such as ethyl hexanoate, ethyl octanoate, and ethyl decanoate (Table 1, Figure S3). At the investigated stage of fermentation, *L. thermotolerans* and *S. pombe* starters produced the highest amounts of ethyl hexanoate and together with *P. kluyveri* were the most abundant in ethyl octanoate, the two among the most important fruity esters [26]. The concentration of ethyl 9-decenoate, another carrier of fruity notes, was higher in *L. thermotolerans* and *S. pombe* than in most other musts. *Lachancea thermotolerans* was previously found to enhance the concentration of ethyl esters in sequential fermentation [10]. It is known that during fermentation the production of linear ethyl esters and other volatile compounds fluctuates, often peaking at the end of the growth phase and decreasing during the stationary phase, sometimes with the second peak corresponding to the start of the decline phase and release of intracellular volatiles after yeast cell autolysis [21]. Various non-*Saccharomyces* yeasts exhibit diverse performance and dynamics of the production of volatile compounds during fermentation, which is among the probable reasons for the differences observed in this study at the monitored point of fermentation. 

Compared to ethyl esters, the concentrations of acetates formed during alcoholic fermentation depend more on yeast enzymatic activity, especially that of acetyltransferases, and less on substrate availability [21]. The highest concentrations of propyl and isobutyl acetate, esters exerting fruity notes, were found in the control treatment inoculated with *S. cerevisiae*, while *M. pulcherrima* must contained the lowest amounts (Table 1, Figure S4). In general, the must inoculated with *P. kluyveri* contained elevated levels of isoamyl and hexyl acetate, two major acetate esters responsible for fruity and flowery notes [26], respectively. *Pichia kluyveri* must was the most abundant in both 3-hexen-1-yl acetate isomers and also the sole treatment with the ratio of *trans* to *cis* form higher than one (Table 1, Figure S4), suggesting a possible activity of particular invertases. *Metschnikowia* *pulcherrima* and especially *T. delbrueckii* starters produced the lowest quantities of the abovementioned fruity acetates. Despite its positive influence on the concentration of particular esters, *M. pulcherrima* was previously shown to be able to reduce the content of acetates [10]. The results obtained for *T. delbrueckii* were partly in agreement with previous studies that observed both a decrease [11,12] and an increase [13,16] in isoamyl acetate concentration after inoculation with this yeast in co-fermentation.

The largest difference among the acetates was observed for 2-phenethyl acetate, an ester imparting pleasant floral notes to wine. It was produced in the highest concentration by *P. kluyveri*, with a two- to three-fold increase relative to the other musts. Such an outcome at this stage corresponded significantly to previous findings showing an increase in the concentration of this ester in finished wines after sequential or mixed inoculation with this non-*Saccharomyces* yeast [10,15,16], suggesting this effect remains significant throughout fermentation. *Schizosaccharomyces pombe* fermented must contained the highest concentration of isobornyl acetate. 

A number of esters of methanol and higher alcohols with various fatty acids were identified (Table 1, Figure S5). While the odor of methyl hexanoate is generally described as fruity, methyl esters of higher molecular weights are perceived as fatty and waxy. *Saccharomyces cerevisiae* and especially *T. delbrueckii* musts contained the lowest concentration of the majority of these esters at this stage of fermentation. Interestingly, *T. delbrueckii* must, previously found to contain an elevated level of ethyl propanoate, was characterized by the highest concentrations of other esters of propanoic acid, such as isoamyl propanoate (fruity) and 2-phenethyl propanoate (floral), the latter not commonly synthesized by *S. cerevisiae* (Table 1, Figure S5). The observed highest concentration of 2-phenethyl propanoate was in accordance with a previous investigation reporting high concentration in *T. delbrueckii* inoculated must [22]. Beckner Whitener et al. [22] found this ester in grape musts fermented by some other non-*Saccharomyces* yeasts as well, such as *M. pulcherrima*, *L. thermotolerans*, and especially *Kazachstania gamospora*. In this study, *M. pulcherrima* also produced a notable concentration of 2-phenethyl propanoate, although lower than that found in *T. delbrueckii* inoculated must, while traces of this ester (*m/z* 104) were detected in musts of the remaining non-*Saccharomyces* treatments (Table 1). Both this and the cited study [22] tentatively identified 2-phenethyl propanoate only in the initial stage of fermentation, so it is yet to be confirmed if it remains detectable in the wine after fermentation completion. No such data were found in the literature. 

Esters of isoamyl alcohol and isobutanol with middle-chain fatty acids were generally lower in *T. delbrueckii* and *S. cerevisiae* treatments (Table 1). As in the case of the majority of minor esters identified, their odor perception thresholds are still unknown, which makes it impossible to assess their potential impact on wine aroma.

#### 3.2.7. Miscellaneous Compounds

The most abundant aldehyde identified was hexanal, a precursor to hexanol and other C_6_-alcohols imparting ´green´ notes. The highest level was observed in *M. pulcherrima*, followed by *S. cerevisiae* must. *Metschnikowia pulcherrima* and *P. kluyveri* contained the highest, and *T. delbrueckii* the lowest level of 3-methylbutanal. *Saccharomyces cerevisiae* produced the highest benzaldehyde level. Among other miscellaneous compounds, the concentration of dihydro-2-methyl-3(2H)-thiophenone differentiated well the majority of the treatments. It was the highest in *T. delbrueckii*, followed by *L. thermotolerans*, then the *M. pulcherrima* and *P. kluyveri* musts, which did not differ from each other; even lower concentrations were recorded in *S. pombe*, while the lowest amount was produced by *S. cerevisiae* yeast (Table 1, Figure S6). The potential of the majority of compounds from this group to affect wine aroma is still unknown, mostly due to a lack of information about their odor perception thresholds.

#### 3.2.8. Multivariate Statistical Analysis

**Figure 2:**
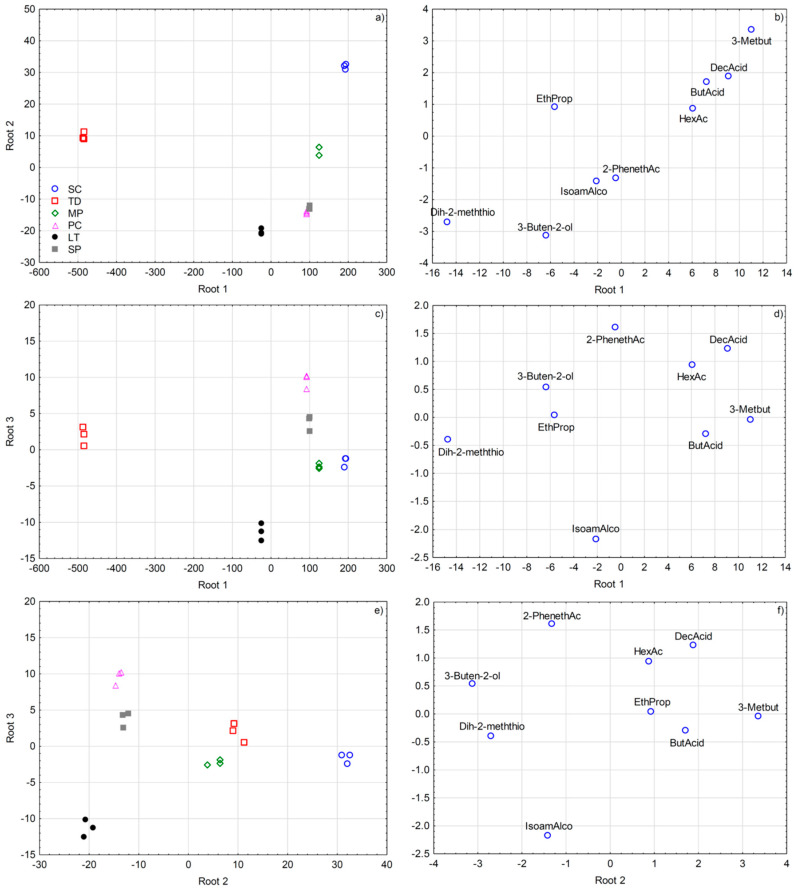
Separation of Malvazija istarska grape musts in early fermentation according to inoculated yeast species defined by the first three discriminant functions (roots) obtained by forward stepwise discriminant analysis on the basis of volatile aroma compound composition. Projection of grape must samples is shown in sub-figures (**a**,**c**,**e**), while standardized coefficients of the variables (volatile aroma compounds) are shown in sub-figures (**b**,**d**,**f**). Abbreviations: SC—*Saccharomyces cerevisiae*; TD—*Torulaspora delbrueckii*; MP—*Metschnikowia pulcherrima*; PC—*Pichia kluyveri*; LT—*Lachancea thermotolerans*; SP—*Schizosaccharomyces pombe*; Dih-2-meththio—dihydro-2-methyl-3(2H)-thiophenone; EthProp—ethyl propanoate; IsoamAlco—isoamyl alcohol; 2-PhenethAc—2-phenethyl acetate; HexAc—hexyl acetate; ButAcid—butanoic acid; DecAcid—decanoic acid; 3-Metbut—3-methylbutanal.

To better visualize the diversity in volatile aroma compound composition produced by the investigated non-*Saccharomyces* yeasts in the early phase of grape must fermentation, and to extract the most useful variables for their differentiation, SLDA was applied on mean-centred data of a reduced dataset including 40 variables (volatile compound concentrations) with the highest *F*-ratios. This SLDA model correctly classified all the grape must samples according to yeast species and extracted the nine most useful variables for their differentiation (Figure 2), with rather high squared Mahalanobis distances from group centroids (numerical data not shown). All the samples (100%) were classified correctly after including only two variables already, ethyl propanoate and 2-phenethyl acetate, while the SLDA model further extracted butyric acid, dihydro-2-methyl-3(2H)-thiophenone, 3-methylbutanal, 3-buten-2-ol, decanoic acid, hexanoic acid, and isoamyl alcohol.

**Figure 3:**
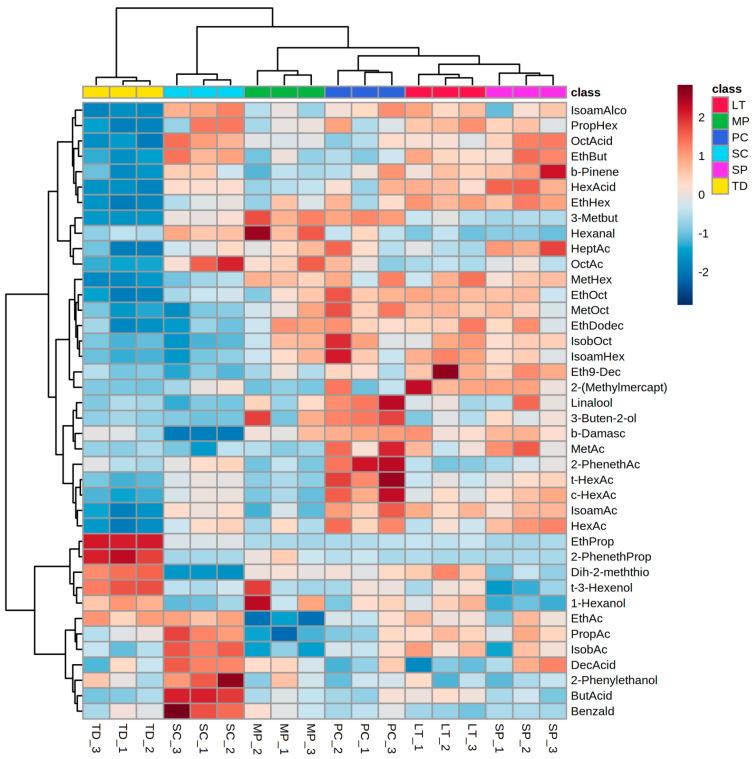
Hierarchical clustering analysis performed using volatile aroma compound composition of Malvazija istarska grape must in early fermentation by *Saccharomyces cerevisiae* and various non-*Saccharomyces* yeasts. The rows in the heatmap represent compounds, and the columns indicate samples. Colours of heatmap cells indicate low (dark blue), medium (white), and high (dark red) abundance of a particular compound. Abbreviations: IsoamAlco—isoamyl alcohol; PropHex—propyl hexanoate; OctAcid—octanoic acid; EthBut—ethyl butyrate; b-Pinene—β-pinene; HexAcid—hexanoic acid; EthHex—ethyl hexanoate; 3-Metbut—3-methylbutanal; HeptAc—heptyl acetate; OctAc—octyl acetate; MetHex—methyl hexanoate; EthOct—ethyl octanoate; MetOct—methyl octanoate; EthDodec—Ethyl dodecanoate; IsobOct—Isobutyl octanoate; IsoamHex—isoamyl hexanoate; Eth9-Dec—ethyl 9-decenoate; 2-(Methylmercapt)—2-(methylmercapto)benzothiazole; b-Damasc—β-damascenone; MetAc—methyl acetate; 2-PhenethAc—2-phenethyl acetate; t-HexAc—*trans*-hexen-1-yl acetate; c-HexAc—*cis*-hexen-1-yl acetate; IsoamAc—isoamyl acetate; HexAc—hexyl acetate; EthProp—ethyl propanoate; 2-PhenethProp—2-phenethyl propanoate; Dih-2-meththio—dihydro-2-methyl-3(2H)-thiophenone; t-3-Hexenol—*trans*-3-hexen-1-ol; EthAc—ethyl acetate; PropAc—propyl acetate; LT—*Lachancea thermotolerans*; MP—*Metschnikowia pulcherrima*; PC—*Pichia kluyveri*; SC—*Saccharomyces cerevisiae*; SP—*Schizosaccharomyces pombe*; TD—*Torulaspora delbrueckii*.

Hierarchical clustering analysis performed on a reduced dataset including 40 variables (volatile compound concentrations) with the highest *F*-ratios, confirmed that each yeast species studied produced a distinct volatile profile in the early phase of fermentation (Figure 3). *Saccharomyces cerevisiae* early ferment was separated from the musts inoculated by non-*Saccharomyces* yeasts mostly by higher concentrations of propyl acetate, isobutyl acetate, butyric acid, decanoic acid, 2-phenylethanol, and benzaldehyde, and the lowest concentrations of compounds such as β-damascenone and dihydro-2-methyl-3(2H)-thiophenone. *Saccharomyces cerevisiae* grape must was also characterized by lower concentrations of particular esters compared to the musts inoculated with non-*Saccharomyces* yeasts except *T. delbrueckii*, such as methyl octanoate, ethyl dodecanoate, isobutyl octanoate, isoamyl hexanoate, and ethyl 9-decenoate. *Torulaspora delbrueckii* must contain the lowest concentrations of a large array of compounds and, at the same time, was distinguished by the highest concentrations of ethyl propanoate, 2-phenethyl propanoate, dihydro-2-methyl-3(2H)-thiophenone, and *trans*-3-hexen-1-ol, which resulted in the largest distance from the other species. *Pichia kluyveri* stood out with increased levels of linalool, 3-buten-2-ol, β-damascenone and a whole range of acetate esters. The highest levels of β-pinene, hexanoic acid, and ethyl hexanoate were characteristic of the *S. pombe* early ferment. Although evidently different and distant from each other, grape musts inoculated with *S. cerevisiae* and *S. pombe* were distinguished from the others by high octanoic acid and ethyl butyrate, and low *trans*-2-hexen-1-ol, 1-hexanol, and dihydro-2-methyl-3(2H)-thiophenone concentrations.

## 4. Conclusions

The results of this study showed that the studied non-*Saccharomyces* yeasts produce diverse volatile aroma profiles in the early phase of monoculture fermentation, significantly different from each other and from that produced by *S. cerevisiae*. Many of the investigated non-*Saccharomyces* yeasts exhibited undoubtedly positive characteristics in this phase of fermentation in quantitative terms, such as increases in linalool and β-damascenone concentrations, lower production of higher alcohols, and improved synthesis of many major and minor odoriferous esters. 

Regarding particular volatile compounds, *T. delbrueckii* produced the highest levels of *trans*-3-hexen-1-ol, ethyl propanoate, ethyl isobutyrate, isoamyl propanoate, 2-phenethyl propanoate, and dihydro-2-methyl-3(2H)-thiophenone, *P. kluyveri* excelled in the production of acetates, particularly *cis*-3-hexen-1-yl acetate, *trans*-3-hexen-1-yl acetate, and 2-phenethyl acetate, while the highest levels of *cis*-2-hexen-1-ol and hexanoic acid were found in must inoculated with *S. pombe*. The *Saccharomyces cerevisiae* control must contained the highest concentrations of 2-phenylethanol, butyric acid, propyl acetate, and isobutyl acetate. Particular yeasts, such as *T. delbrueckii* and *M. pulcherrima* synthesized esters not commonly found in *S. cerevisiae* fermented wines, such as 2-phenethyl propanoate. However, it is yet to be established if and to what degree these contributions persist after sequential inoculation with *S. cerevisiae*, which certainly varies depending on the biocompatibility of the co-fermenting yeasts in diverse grape must matrices. Our group is currently working on this topic.

## Figures and Tables

**Table 1:** Concentrations (μg/L)* of volatile aroma compounds identified in the early phase of fermentation of the Malvazija istarska grape must inoculated by *Saccharomyces cerevisiae* and five non-*Saccharomyces* yeasts obtained by headspace solid-phase microextraction and gas chromatography-mass spectrometry.

Volatile Compound	ID	LRI_exp_	LRI_lit_	Yeast Species					
				*Saccharomyces cerevisiae*	*Torulaspora delbrueckii*	*Metschnikowia pulcherrima*	*Pichia kluyveri*	*Lachancea thermotolerans*	*Schizosacchaomyces pombe*
*Terpenes*									
Camphene	MS, LRI	1056	1056	0.024 ± 0.006 ^b^	0.024 ± 0.005 ^b^	0.023 ± 0.006 ^b^	0.039 ± 0.019 ^b^	0.041 ± 0.015 ^b^	0.093 ± 0.051 ^a^
β-Pinene	MS, LRI	1146	1145	0.16 ± 0.01 ^bc^	0.13 ± 0.00 ^d^	0.14 ± 0.01 ^cd^	0.16 ± 0.01 ^bc^	0.16 ± 0.00 ^ab^	0.17 ± 0.01 ^a^
Limonene	MS, LRI	1191	1196	0.30 ± 0.04	0.35 ± 0.08	0.39 ± 0.18	0.28 ± 0.03	0.25 ± 0.02	0.27 ± 0.02
β-Phellandrene	MS, LRI	1208	1218	0.068 ± 0.004	0.070 ± 0.012	0.064 ± 0.002	0.064 ± 0.008	0.061 ± 0.015	0.064 ± 0.003
Eucalyptol	MS, LRI	1216	1224	0.061 ± 0.004 ^b^	0.092 ± 0.028 ^a^	0.071 ± 0.014 ^ab^	0.067 ± 0.018 ^ab^	0.055 ± 0.018 ^b^	0.067 ± 0.002 ^ab^
Menthol	MS, LRI	1637	1641	1.55 ± 0.10 ^b^	1.48 ± 0.23 ^b^	1.30 ± 0.20 ^b^	2.55 ± 1.42 ^ab^	2.01 ± 0.64 ^b^	4.97 ± 3.02 ^a^
6,10-Dihydromyrcenol	MS, LRI	1473	1475	0.09 ± 0.01 ^c^	0.12 ± 0.01 ^bc^	0.12 ± 0.02 ^bc^	0.16 ± 0.06 ^ab^	0.11 ± 0.05 ^bc^	0.18 ± 0.03 ^a^
Linalool	S, MS, LRI	1542	1542	3.76 ± 0.08 ^c^	3.90 ± 0.07 ^bc^	4.15 ± 0.22 ^bc^	4.75 ± 0.24 ^a^	4.05 ± 0.12 ^bc^	4.27 ± 0.39 ^b^
α-Farnesene	MS, LRI	1752	1762	0.037 ± 0.011	0.032 ± 0.004	0.031 ± 0.007	0.022 ± 0.013	0.033 ± 0.010	0.030 ± 0.016
Geranyl acetate	MS, LRI	1764	1768	0.099 ± 0.029 ^ab^	0.049 ± 0.007 ^c^	0.067 ± 0.011 ^bc^	0.079 ± 0.028 ^abc^	0.108 ± 0.021 ^a^	0.111 ± 0.027 ^a^
Geranyl acetone	MS, LRI	1849	1845	0.17 ± 0.04	0.40 ± 0.18	0.39 ± 0.33	0.34 ± 0.14	0.22 ± 0.02	0.41 ± 0.25
*C_13_-norisoprenoids*									
β-Damascenone	MS, LRI	1809	1809	0.35 ± 0.00 ^d^	0.50 ± 0.03 ^c^	0.54 ± 0.04 ^bc^	0.61 ± 0.01 ^a^	0.57 ± 0.05 ^ab^	0.58 ± 0.03 ^ab^
α-Isomethylionone	MS, LRI	1835	1848	0.24 ± 0.03 ^ab^	0.20 ± 0.01 ^b^	0.23 ± 0.02 ^ab^	0.68 ± 0.45 ^ab^	0.43 ± 0.32 ^ab^	0.70 ± 0.42 ^a^
β-Ionone	MS, LRI	1916	1915	0.20 ± 0.02 ^ab^	0.18 ± 0.01 ^b^	0.20 ± 0.01 ^ab^	0.52 ± 0.33 ^ab^	0.33 ± 0.22 ^ab^	0.54 ± 0.28 ^a^
β-Methylionone	MS, LRI	2012	1988	1.87 ± 0.11	1.40 ± 0.14	1.46 ± 0.19	4.89 ± 3.52	2.89 ± 2.12	4.83 ± 2.56
6-Methylionone	MS	2098	n/a	0.19 ± 0.02 ^abc^	0.15 ± 0.01 ^c^	0.16 ± 0.02 ^bc^	0.38 ± 0.21 ^ab^	0.24 ± 0.15 ^abc^	0.41 ± 0.18 ^a^
*Alcohols*									
3-Buten-2-ol	MS, LRI	1051	NA	0.19 ± 0.01 ^c^	0.23 ± 0.01 ^c^	0.43 ± 0.22 ^ab^	0.58 ± 0.05 ^a^	0.26 ± 0.06 ^bc^	0.35 ± 0.04 ^bc^
Isobutanol	MS, LRI	1090	1098	5.50 ± 0.52 ^abc^	4.71 ± 0.11 ^c^	4.94 ± 0.45 ^bc^	5.54 ± 1.23 ^abc^	6.30 ± 0.67 ^a^	6.19 ± 1.00 ^ab^
Isoamyl alcohol	MS, LRI	1229	1229	328.6 ± 9.9 ^a^	213.5 ± 3.2 ^d^	269.6 ± 14.01 ^c^	307.9 ± 24.1 ^ab^	313.1 ± 13.0 ^ab^	281.1 ± 35.4 ^bc^
1-Hexanol	S, MS, LRI	1357	1357	1107.0 ± 33.5 ^b^	1336.0 ± 35.3 ^a^	1359.0 ± 179.6 ^a^	1213.8 ± 94.3 ^ab^	1273.7 ± 62.9 ^a^	1072.2 ± 20.3 ^b^
*trans*-3-Hexen-1-ol	S, MS, LRI	1366	1361	63.02 ± 1.19 ^bc^	78.85 ± 1.78 ^a^	68.22 ± 11.29 ^b^	64.91 ± 2.90 ^bc^	65.22 ± 3.25 ^bc^	57.13 ± 3.42 ^c^
*cis*-3-Hexen-1-ol	S, MS, LRI	1389	1389	70.51 ± 5.11 ^ab^	75.03 ± 1.69 ^a^	75.09 ± 6.97 ^a^	69.15 ± 7.52 ^ab^	66.13 ± 5.80 ^ab^	61.48 ± 2.78 ^b^
*cis*-2-Hexen-1-ol	MS, LRI	1416	1413	0.10 ± 0.02 ^b^	0.08 ± 0.01 ^b^	0.11 ± 0.01 ^ab^	0.09 ± 0.01 ^b^	0.08 ± 0.01 ^b^	0.13 ± 0.03 ^a^
6-Methyl-5-hepten-2-ol	MS, LRI	1463	1466	0.22 ± 0.03 ^bc^	0.26 ± 0.01 ^a^	0.23 ± 0.02 ^abc^	0.25 ± 0.02 ^ab^	0.23 ± 0.02 ^abc^	0.21 ± 0.01 ^c^
2-Phenylethanol	S, MS, LRI	1891	1893	5053.5 ± 743.7 ^a^	3371.6 ± 517.4 ^b^	3278.1 ± 663.2 ^b^	2924.3 ± 438.0 ^b^	3004.9 ± 670.3 ^b^	2791.9 ± 354.7 ^b^
*Volatile acids*									
Acetic acid	MS, LRI	1445	1439	10.42 ± 2.17 ^ab^	8.12 ± 1.94 ^b^	8.76 ± 0.60 ^ab^	11.44 ± 2.16 ^a^	10.66 ± 2.10 ^ab^	8.89 ± 1.12 ^ab^
Butyric acid	S, MS, LRI	1617	1612	970.6 ± 21.8 ^a^	499.1 ± 38.1 ^c^	570.6 ± 33.8 ^bc^	560.8 ± 79.1 ^c^	645.4 ± 19.8 ^b^	530.4 ± 56.3 ^c^
Hexanoic acid	S, MS, LRI	1824	1828	3070.9 ± 24.4 ^b^	1879.9 ± 72.1 ^d^	2609.9 ± 105.9 ^c^	2991.7 ± 487.6 ^bc^	3298.9 ± 219.4 ^b^	3768.8 ± 272.6 ^a^
Octanoic acid	S, MS, LRI	2043	2042	3878.1 ± 187.6 ^a^	2347.1 ± 120.0 ^c^	3213.4 ± 23.8 ^b^	3092.8 ± 304.0 ^b^	3312.5 ± 88.4 ^b^	3847.4 ± 288.5 ^a^
Nonanoic acid	MS, LRI	2155	2119	63.60 ± 15.87 ^a^	59.29 ± 6.65 ^ab^	58.67 ± 8.51 ^ab^	26.12 ± 29.93 ^bc^	42.46 ± 31.64 ^ab^	7.74 ± 0.54 ^c^
Decanoic acid	S, MS, LRI	2257	2258	1874.6 ± 64.6 ^a^	1349.3 ± 220.0 ^bc^	1506.3 ± 94.3 ^b^	1322.4 ± 261.6 ^bc^	1107.6 ± 134.7 ^c^	1622.8 ± 277.3 ^ab^
*Ethyl esters*									
Ethyl acetate	MS, LRI	<1000	885	96.29 ± 4.21 ^a^	96.16 ± 7.77 ^a^	44.00 ± 7.12 ^c^	77.64 ± 5.72 ^b^	85.32 ± 7.35 ^ab^	79.22 ± 15.74 ^b^
Ethyl propanoate	MS, LRI	<1000	949	0.20 ± 0.01 ^b^	0.87 ± 0.01 ^a^	0.07 ± 0.01 ^e^	0.10 ± 0.02 ^d^	0.14 ± 0.01 ^c^	0.07 ± 0.01 ^e^
Ethyl isobutyrate	MS, LRI	<1000	965	0.01 ± 0.00 ^b^	0.02 ± 0.00 ^a^	0.01 ± 0.00 ^b^	0.01 ± 0.01 ^ab^	0.01 ± 0.00 ^b^	0.01 ± 0.00 ^b^
Ethyl butyrate	S, MS, LRI	1030	1030	81.37 ± 7.02 ^a^	30.04 ± 3.65 ^c^	48.17 ± 10.95 ^b^	48.58 ± 6.44 ^b^	70.07 ± 7.51 ^a^	79.58 ± 14.44 ^a^
Ethyl hexanoate	S, MS, LRI	1242	1236	678.4 ± 49.5 ^b^	363.9 ± 35.8 ^c^	706.2 ± 134.6 ^b^	756.6 ± 143.3 ^b^	921.6 ± 39.2 ^a^	924.4 ± 75.5 ^a^
Ethyl octanoate	S, MS, LRI	1435	1435	1744.0 ± 132.2 ^b^	779.2 ± 193.8 ^c^	1981.0 ± 525.5 ^b^	2738.0 ± 432.3 ^a^	2599.5 ± 83.1 ^a^	2265.1 ± 400.8 ^ab^
Ethyl nonanoate	MS, LRI	1530	1535	6.14 ± 0.54 ^a^	4.54 ± 0.67 ^b^	5.37 ± 0.27 ^ab^	5.84 ± 0.56 ^a^	5.44 ± 0.83 ^ab^	5.93 ± 0.56 ^a^
Ethyl 2-furoate	MS, LRI	1609	1606	0.022 ± 0.002 ^ab^	0.021 ± 0.005 ^ab^	0.025 ± 0.006 ^a^	0.014 ± 0.012 ^b^	0.018 ± 0.004 ^ab^	0.022 ± 0.004 ^ab^
Ethyl decanoate	S, MS, LRI	1645	1638	1192.0 ± 167.1 ^ab^	1038.2 ± 132.9 ^b^	1467.3 ± 336.7 ^a^	1499.1 ± 199.3 ^a^	1571.7 ± 244.7 ^a^	1563.4 ± 269.3 ^a^
Ethyl 9-decenoate	MS, LRI	1694	1688	0.64 ± 0.32 ^d^	1.09 ± 0.35 ^cd^	1.64 ± 0.48 ^cd^	2.12 ± 0.61 ^bc^	3.89 ± 1.52 ^a^	3.19 ± 0.53 ^ab^
Ethyl dodecanoate	MS, LRI	1843	1843	56.14 ± 9.48 ^b^	49.71 ± 14.43 ^b^	95.08 ± 17.94 ^a^	94.53 ± 11.58 ^a^	98.13 ± 12.49 ^a^	91.20 ± 16.02 ^a^
*Acetate esters*									
Methyl acetate	MS, LRI	<1000	813	0.14 ± 0.02 ^b^	0.15 ± 0.00 ^b^	0.15 ± 0.01 ^b^	0.22 ± 0.04 ^a^	0.18 ± 0.02 ^ab^	0.20 ± 0.03 ^a^
Propyl acetate	MS, LRI	<1000	982	1.34 ± 0.09 ^a^	0.99 ± 0.05 ^bc^	0.69 ± 0.11 ^d^	0.94 ± 0.14 ^c^	1.13 ± 0.05 ^b^	1.12 ± 0.12 ^b^
Isobutyl acetate	S, MS, LRI	1015	1009	89.76 ± 2.57 ^a^	60.13 ± 5.31 ^cd^	52.15 ± 5.24 ^d^	66.99 ± 3.82 ^bc^	77.25 ± 6.30 ^ab^	65.71 ± 14.23 ^bc^
Butyl acetate	MS, LRI	1062	1064	0.19 ± 0.05 ^ab^	0.15 ± 0.01 ^b^	0.14 ± 0.02 ^b^	0.19 ± 0.03 ^ab^	0.22 ± 0.01 ^a^	0.23 ± 0.05 ^a^
Isoamyl acetate	S, MS, LRI	1133	1133	1893.4 ± 50.2 ^b^	1188.6 ± 91.2 ^d^	1494.3 ± 219.2 ^c^	2231.5 ± 223.6 ^a^	2084.3 ± 112.1 ^ab^	2028.5 ± 142.8 ^ab^
Hexyl acetate	S, MS, LRI	1272	1272	486.0 ± 29.5 ^b^	340.1 ± 17.4 ^c^	452.3 ± 41.1 ^b^	556.3 ± 45.0 ^a^	459.1 ± 23.2 ^b^	554.4 ± 24.03 ^a^
*cis*-3-Hexen-1-yl acetate	MS, LRI	1304	1300	2.73 ± 0.09 ^c^	1.66 ± 0.09 ^d^	2.04 ± 0.24 ^d^	4.20 ± 0.63 ^a^	2.78 ± 0.21 ^bc^	3.30 ± 0.31 ^b^
*trans*-3-Hexen-1-yl acetate	MS, LRI	1313	1316	2.78 ± 0.09 ^b^	1.53 ± 0.18 ^d^	2.0 ± 0.16 ^cd^	5.21 ± 0.86 ^a^	2.59 ± 0.20 ^bc^	3.13 ± 0.28 ^b^
Heptyl acetate	MS, LRI	1374	1374	0.085 ± 0.009 ^b^	0.043 ± 0.014 ^c^	0.094 ± 0.011 ^ab^	0.097 ± 0.027 ^ab^	0.075 ± 0.007 ^b^	0.119 ± 0.013 ^a^
Octyl acetate	MS, LRI	1481	1483	0.21 ± 0.07 ^a^	0.02 ± 0.00 ^d^	0.17 ± 0.06 ^ab^	0.12 ± 0.06 ^bc^	0.08 ± 0.01 ^cd^	0.11 ± 0.02 ^bc^
Isobornyl acetate	MS, LRI	1570	1571	1.65 ± 0.10 ^b^	1.43 ± 0.30 ^b^	1.40 ± 0.32 ^b^	2.71 ± 1.43 ^b^	2.66 ± 0.67 ^b^	6.74 ± 4.54 ^a^
2-Phenethyl acetate	S, MS, LRI	1803	1801	101.80 ± 10.66 ^b^	74.36 ± 12.22 ^bc^	57.37 ± 15.66 ^c^	187.70 ± 24.84 ^a^	58.27 ± 13.48 ^c^	79.33 ± 13.59 ^bc^
*Other esters*									
Methyl hexanoate	MS, LRI	1170	1172	0.60 ± 0.04 ^b^	0.47 ± 0.02 ^c^	0.79 ± 0.03 ^a^	0.79 ± 0.12 ^a^	0.79 ± 0.12 ^a^	0.76 ± 0.04 ^a^
Isoamyl propanoate	MS, LRI	1179	1181	0.020 ± 0.017 ^ab^	0.040 ± 0.003 ^a^	0.015 ± 0.021 ^b^	0.013 ± 0.003 ^b^	0.005 ± 0.001 ^b^	0.014 ± 0.020 ^b^
Isoamyl butyrate	MS, LRI	1262	1266	0.050 ± 0.021 ^ab^	0.036 ± 0.004 ^b^	0.043 ± 0.005 ^ab^	0.057 ± 0.026 ^ab^	0.064 ± 0.024 ^ab^	0.073 ± 0.004 ^a^
Propyl hexanoate	MS, LRI	1324	1319	0.107 ± 0.037 ^a^	0.033 ± 0.008 ^b^	0.079 ± 0.011 ^a^	0.088 ± 0.024 ^a^	0.110 ± 0.010 ^a^	0.095 ± 0.012 ^a^
Methyl 2-methyloctanoate	MS, LRI	1399	1380	11.27 ± 0.77	10.53 ± 0.86	10.52 ± 0.28	10.66 ± 0.24	10.16 ± 1.42	11.56 ± 1.00
Methyl octanoate	MS, LRI	1407	1404	1.27 ± 0.50 ^c^	1.13 ± 0.34 ^c^	2.78 ± 0.68 ^b^	3.79 ± 0.72 ^a^	3.22 ± 0.15 ^ab^	2.85 ± 0.50 ^b^
Isoamyl hexanoate	MS, LRI	1457	1458	0.33 ± 0.15 ^b^	0.30 ± 0.05 ^b^	0.82 ± 0.20 ^a^	1.02 ± 0.46 ^a^	1.11 ± 0.08 ^a^	0.80 ± 0.11 ^a^
Propyl octanoate	MS, LRI	1520	1510	0.16 ± 0.07 ^a^	0.07 ± 0.02 ^b^	0.16 ± 0.04 ^a^	0.18 ± 0.08 ^a^	0.20 ± 0.02 ^a^	0.17 ± 0.03 ^a^
Isobutyl octanoate	MS, LRI	1550	1551	0.030 ± 0.008 ^b^	0.037 ± 0.005 ^b^	0.082 ± 0.019 ^a^	0.100 ± 0.034 ^a^	0.090 ± 0.020 ^a^	0.082 ± 0.005 ^a^
Methyl decanoate	MS, LRI	1594	1593	0.34 ± 0.07 ^b^	0.33 ± 0.05 ^b^	0.57 ± 0.11 ^a^	0.58 ± 0.15 ^a^	0.60 ± 0.07 ^a^	0.56 ± 0.09 ^a^
Diethyl succinate	MS, LRI	1677	1669	3.00 ± 0.31 ^b^	6.98 ± 1.61 ^a^	7.11 ± 3.29 ^a^	5.08 ± 0.47 ^ab^	6.90 ± 2.65 ^a^	5.61 ± 0.90 ^ab^
Ester *m/z* 131, 43, 70, 113	n.i.	1713	n/a	1.66 ± 0.11 ^a^	1.21 ± 0.21 ^b^	1.45 ± 0.15 ^ab^	1.53 ± 0.09 ^a^	1.43 ± 0.30 ^ab^	1.69 ± 0.08 ^a^
Isobutyl decanoate	MS, LRI	1774	1756	0.007 ± 0.005 ^c^	0.014 ± 0.003 ^bc^	0.022 ± 0.005 ^ab^	0.023 ± 0.003 ^a^	0.017 ± 0.006 ^ab^	0.020 ± 0.005 ^ab^
Isobutyl 4-ethylbenzoate	MS, LRI	1788	n/a	0.38 ± 0.07 ^ab^	0.14 ± 0.12 ^c^	0.39 ± 0.16 ^ab^	0.54 ± 0.19 ^a^	0.40 ± 0.13 ^ab^	0.29 ± 0.08 ^bc^
Isoamyl decanoate	MS, LRI	1859	1856	2.15 ± 0.51	1.86 ± 0.18	2.19 ± 0.15	2.28 ± 0.13	2.19 ± 0.31	2.17 ± 0.24
2-Phenethyl propanoate	MS, LRI	1872	1880	n.d.	0.930 ± 0.084 ^a^	0.233 ± 0.131 ^b^	0.057 ± 0.042 ^c^	0.023 ± 0.013 ^c^	0.018 ± 0.004 ^c^
Hexyl salicylate	MS, LRI	2186	2206	0.43 ± 0.06 ^ab^	0.26 ± 0.02 ^b^	0.27 ± 0.02 ^b^	0.47 ± 0.22 ^a^	0.41 ± 0.07 ^ab^	0.59 ± 0.10 ^a^
*Miscellaneous*									
3-Methylbutanal	MS, LRI	<1000	901	0.099 ± 0.005 ^b^	0.024 ± 0.002 ^d^	0.157 ± 0.022 ^a^	0.147 ± 0.006 ^a^	0.083 ± 0.009 ^bc^	0.071 ± 0.003 ^c^
Hexanal	MS, LRI	1068	1070	9.39 ± 0.55 ^b^	5.73 ± 0.53 ^c^	12.08 ± 2.75 ^a^	7.02 ± 1.24 ^bc^	5.19 ± 0.99 ^c^	4.87 ± 0.33 ^c^
2-Octanone	MS, LRI	1279	1284	0.33 ± 0.01 ^a^	0.34 ± 0.03 ^a^	0.34 ± 0.04 ^a^	0.34 ± 0.06 ^a^	0.31 ± 0.01 ^ab^	0.26 ± 0.02 ^b^
Benzaldehyde	S, MS, LRI	1500	1505	1.94 ± 0.35 ^a^	0.88 ± 0.16 ^bc^	0.94 ± 0.12 ^b^	0.80 ± 0.12 ^bc^	0.61 ± 0.09 ^c^	0.71 ± 0.02 ^bc^
Dihydro-2-methyl-3(2H)-thiophenone	MS, LRI	1512	1506	0.41 ± 0.01 ^e^	1.11 ± 0.04 ^a^	0.79 ± 0.02 ^c^	0.80 ± 0.05 ^c^	0.96 ± 0.10 ^b^	0.61 ± 0.08 ^d^
Benzothiazole	MS, LRI	1930	1937	0.36 ± 0.06 ^b^	0.38 ± 0.03 ^ab^	0.40 ± 0.02 ^ab^	0.42 ± 0.07 ^ab^	0.49 ± 0.08 ^a^	0.46 ± 0.06 ^ab^
2-(Methylmercapto) benzothiazole	MS, LRI	2433	2422	1.82 ± 0.50 ^bc^	0.85 ± 0.06 ^c^	0.88 ± 0.14 ^c^	2.06 ± 1.59 ^bc^	3.77 ± 1.10 ^a^	2.90 ± 0.70 ^ab^
Homosalate	MS	2577	n/a	0.054 ± 0.010	0.070 ± 0.013	0.075 ± 0.026	0.103 ± 0.042	0.065 ± 0.043	0.098 ± 0.039
*p*-tert-Amylphenol	MS	2776	n/a	1.18 ± 0.18 ^b^	1.51 ± 0.26 ^ab^	1.71 ± 0.18 ^a^	1.16 ± 0.42 ^b^	1.14 ± 0.12 ^b^	1.07 ± 0.27 ^b^

ID—type of identification; S—retention time and mass spectrum consistent with those of a pure standard and NIST05 mass spectral library; LRI—linear retention index consistent with that found in literature; MS—mass spectrum consistent with a spectrum from NIST05 mass spectral library or literature. Concentrations of compounds without symbol S in the ID column are reported as equivalents of an internal standard via semi-quantification: terpenes and C_13_-norisoprenoids as 1-nonanol, acids as heptanoic acid, and others as 2-octanol equivalents, assuming a response factor = 1. Only MS symbol in the ID column = tentative identification. LRI_exp_—experimental linear retention index; LRI_lit_—linear retention index from literature. Different superscript lowercase letters in a row represent statistically significant differences between the mean values at *p* < 0.05 determined by one-way ANOVA and least significant difference (LSD) test. Other abbreviations: n/a—not available; n.i.—not identified; n.d.—not detected.

## Data Availability

Data is contained within the article or supplementary material.

## Supplementary Materials

The following supporting information can be downloaded at: https://www.mdpi.com/article/10.3390/foods11193088/s1, Figure S1: Concentrations of selected monoterpenes and C_13_–norisoprenoids with high differentiating ability among yeasts (high *F*-ratios obtained by one-way ANOVA) identified in the early phase of fermentation of Malvazija istarska grape must inoculated by *Saccharomyces cerevisiae* and five non-*Saccharomyces* yeasts. Abbreviations: SC—*Saccharomyces cerevisiae*; TD—*Torulaspora delbrueckii*; MP—*Metschnikowia pulcherrima*; PC—*Pichia kluyveri*; LT—*Lachancea thermotolerans*; SP—*Schizosaccharomyces pombe*; Figure S2: Concentrations of selected alcohols and acids with high differentiating ability among yeasts (high *F*-ratios obtained by one-way ANOVA) identified in the early phase of fermentation of Malvazija istarska grape must inoculated by *Saccharomyces cerevisiae* and five non-*Saccharomyces* yeasts. Abbreviations: SC—*Saccharomyces cerevisiae*; TD—*Torulaspora delbrueckii*; MP—*Metschnikowia pulcherrima*; PC—*Pichia kluyveri*; LT—*Lachancea thermotolerans*; SP—*Schizosaccharomyces pombe*; Figure S3: Concentrations of ethyl esters with high differentiating ability among yeasts (high *F*-ratios obtained by one-way ANOVA) identified in the early phase of fermentation of Malvazija istarska grape must inoculated by *Saccharomyces cerevisiae* and five non-*Saccharomyces* yeasts. Abbreviations: SC—*Saccharomyces cerevisiae*; TD—*Torulaspora delbrueckii*; MP—*Metschnikowia pulcherrima*; PC—*Pichia kluyveri*; LT—*Lachancea thermotolerans*; SP—*Schizosaccharomyces pombe*; Figure S4: Concentrations of acetate esters with high differentiating ability among yeasts (high *F*-ratios obtained by one-way ANOVA) identified in the early phase of fermentation of Malvazija istarska grape must inoculated by *Saccharomyces cerevisiae* and five non-*Saccharomyces* yeasts. Abbreviations: SC—*Saccharomyces cerevisiae*; TD—*Torulaspora delbrueckii*; MP—*Metschnikowia pulcherrima*; PC—*Pichia kluyveri*; LT—*Lachancea thermotolerans*; SP—*Schizosaccharomyces pombe*; Figure S5: Concentrations of other esters with high differentiating ability among yeasts (high *F*-ratios obtained by one-way ANOVA) identified in the early phase of fermentation of Malvazija istarska grape must inoculated by *Saccharomyces cerevisiae* and five non-*Saccharomyces* yeasts. Abbreviations: SC—*Saccharomyces cerevisiae*; TD—*Torulaspora delbrueckii*; MP—*Metschnikowia pulcherrima*; PC—*Pichia kluyveri*; LT—*Lachancea thermotolerans*; SP—*Schizosaccharomyces pombe*; Figure S6: Concentrations of miscellaneous volatile compounds with high differentiating ability among yeasts (high *F*-ratios) identified in the early phase of fermentation of Malvazija istarska grape must inoculated by *Saccharomyces cerevisiae* and five non-*Saccharomyces* yeasts. Abbreviations: SC—*Saccharomyces cerevisiae*; TD—*Torulaspora delbrueckii*; MP—*Metschnikowia pulcherrima*; PC—*Pichia kluyveri*; LT—*Lachancea thermotolerans*; SP—*Schizosaccharomyces pombe*.
