# Supplementary material for: Diversity of Volatile Aroma Compound Composition Produced by Non-Saccharomyces Yeasts in the Early Phase of Grape Must Fermentation
Source: Foods. 2022 Oct 5;11(19):3088. doi: 10.3390/foods11193088 (PMC9563004; doi:10.3390/foods11193088)
Supplement: Supplementary file 1 [file foods-11-03088-s001.zip › foods-1901690-supplementary.pdf]

Diversity of volatile aroma compound composition produced by non-*Saccharomyces* yeasts in early phase of grape must fermentation

Doris Delač Salopek [1], Ivana Horvat [1], Ana Hranilović [2], Tomislav Plavša [1], Sanja Radeka [1], Igor Pasković [1], Igor Lukić [1,3,*]

[1] Institute of agriculture and tourism, Karla Huguesa 8, Poreč 52440, Croatia
[2] Department of Wine Science, The University of Adelaide, Urrbrae, SA, 5064, Australia

[3] Centre of Excellence for Biodiversity and Molecular Plant Breeding, Svetošimunska 25, 10000 Zagreb, Croatia
* Correspondence: igor@iptpo.hr; Tel.: 0038552408327

Supplementary Materials

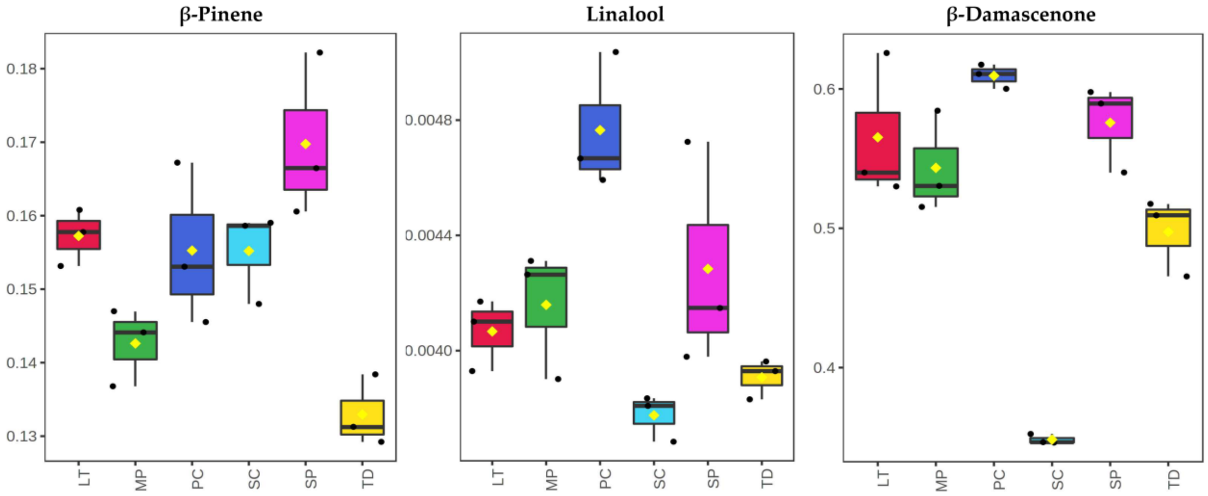

Figure S1. Concentrations of selected monoterpenes and C_{13}–norisoprenoids with high differentiating ability among yeasts (high *F*-ratios) identified in the early phase of fermentation of Malvazija istarska grape must inoculated by *Saccharomyces cerevisiae* and five non-*Saccharomyces* yeasts. Abbreviations: SC – *Saccharomyces cerevisiae*; TD – *Torulaspora delbrueckii*; MP – *Metschnikowia pulcherrima*; PC – *Pichia kluyveri*; LT – *Lachancea thermotolerans*; SP – *Schizosaccharomyces pombe*.

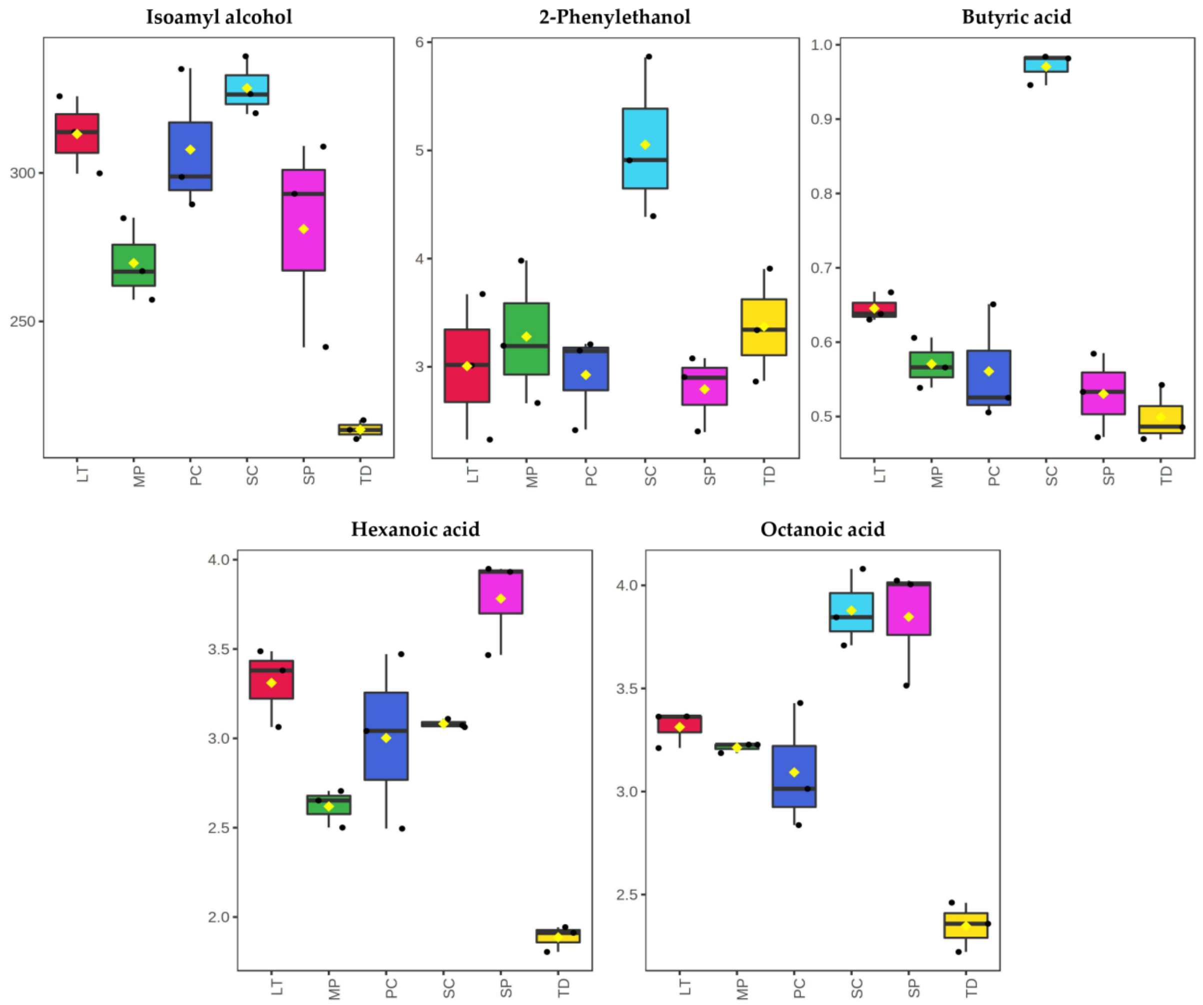

Figure S2. Concentrations of selected alcohols and acids with high differentiating ability among yeasts (high *F*-ratios) identified in the early phase of fermentation of Malvazija istarska grape must inoculated by *Saccharomyces cerevisiae* and five non-*Saccharomyces* yeasts. Abbreviations: SC – *Saccharomyces cerevisiae*; TD – *Torulaspora delbrueckii*; MP – *Metschnikowia pulcherrima*; PC – *Pichia kluyveri*; LT – *Lachancea thermotolerans*; SP – *Schizosaccharomyces pombe*.

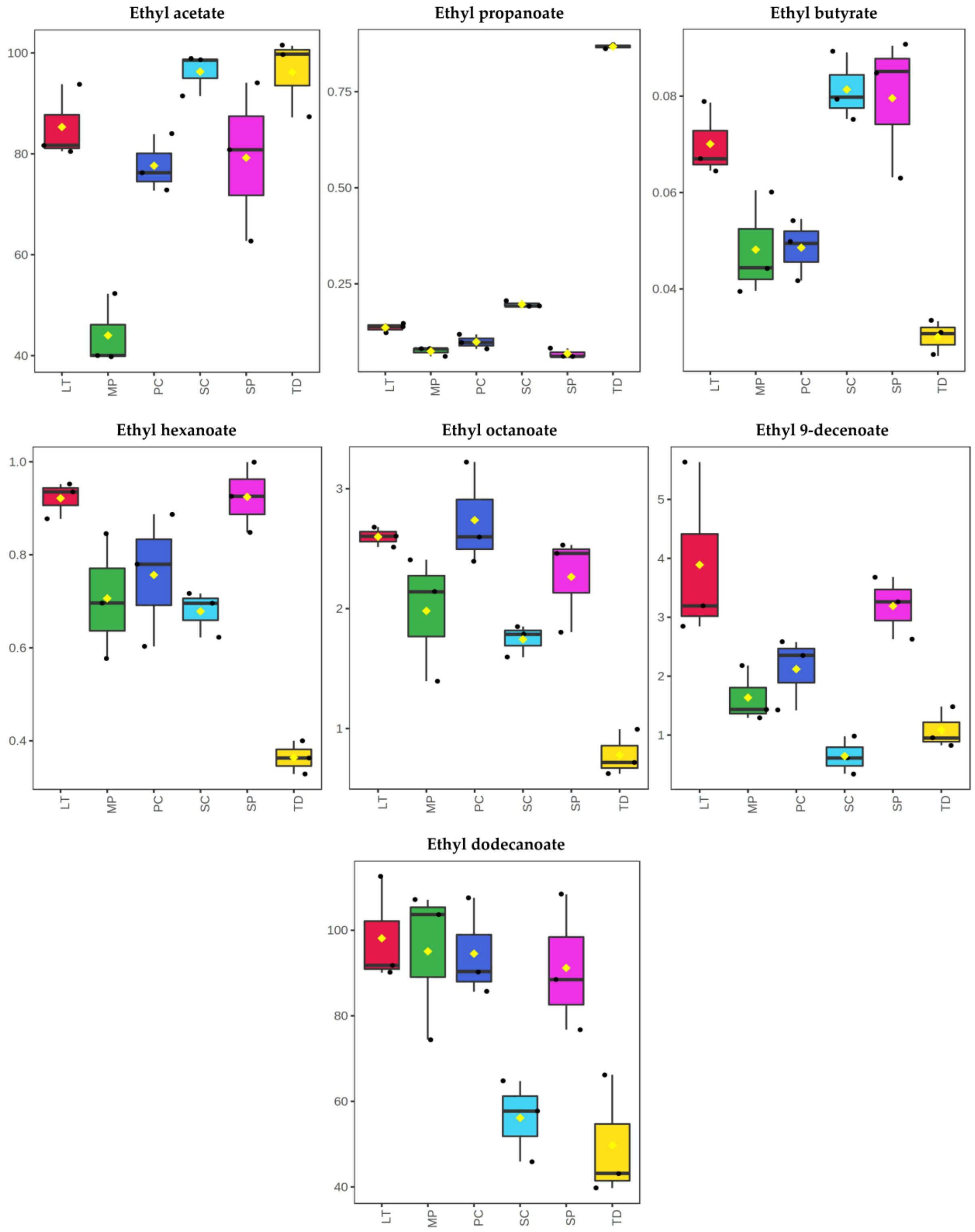

Figure S3. Concentrations of ethyl esters with high differentiating ability among yeasts (high *F*-ratios) identified in the early phase of fermentation of Malvazija istarska grape must inoculated by *Saccharomyces cerevisiae* and five non-*Saccharomyces* yeasts. Abbreviations: SC – *Saccharomyces cerevisiae*; TD – *Torulaspora delbrueckii*; MP – *Metschnikowia pulcherrima*; PC – *Pichia kluyveri*; LT – *Lachancea thermotolerans*; SP – *Schizosaccharomyces pombe*.

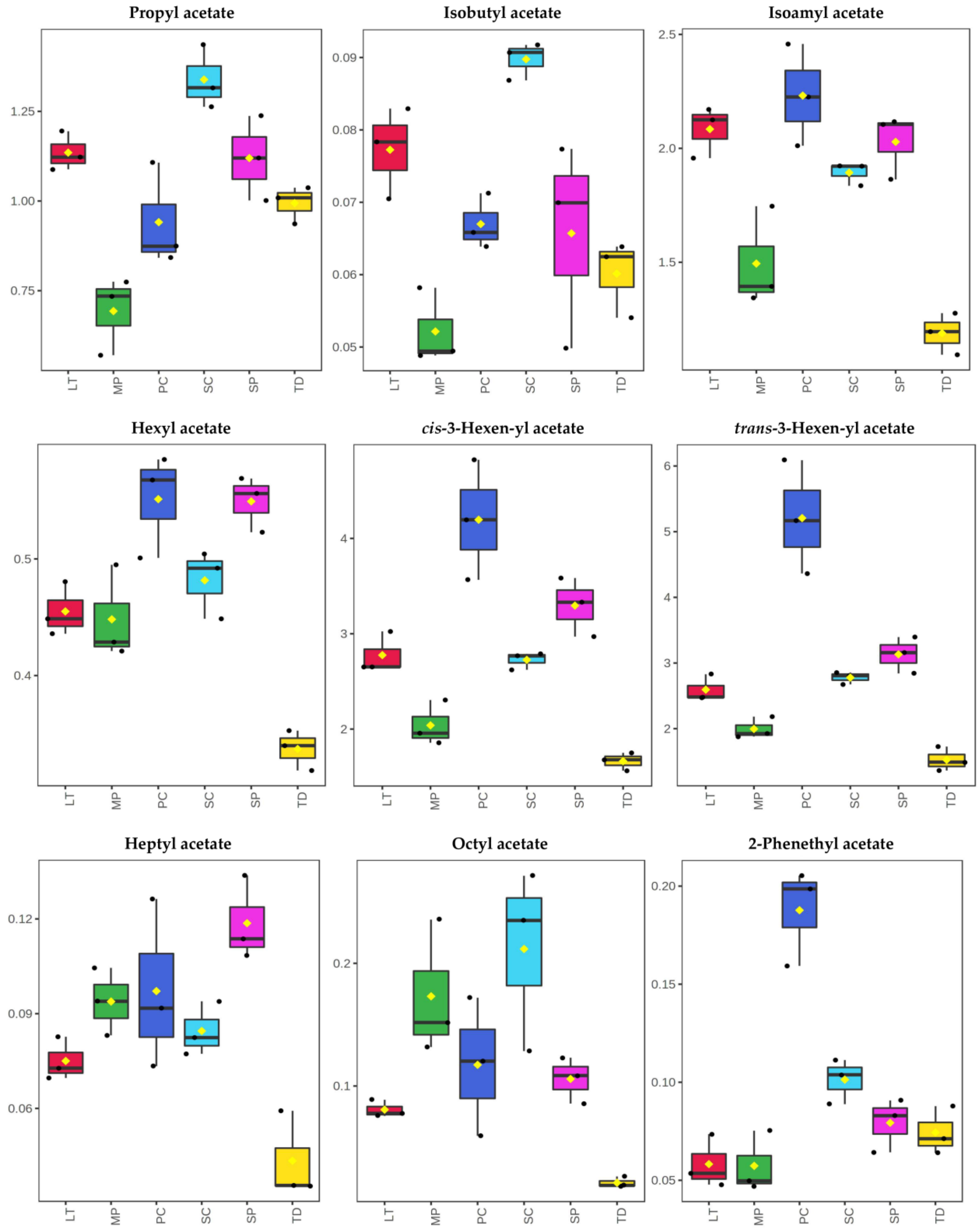

Figure S4. Concentrations of acetate esters with high differentiating ability among yeasts (high *F*-ratios) identified in the early phase of fermentation of Malvazija istarska grape must inoculated by *Saccharomyces cerevisiae* and five non-*Saccharomyces* yeasts. Abbreviations: SC – *Saccharomyces cerevisiae*; TD – *Torulaspora delbrueckii*; MP – *Metschnikowia pulcherrima*; PC – *Pichia kluyveri*; LT – *Lachancea thermotolerans*; SP – *Schizosaccharomyces pombe*.

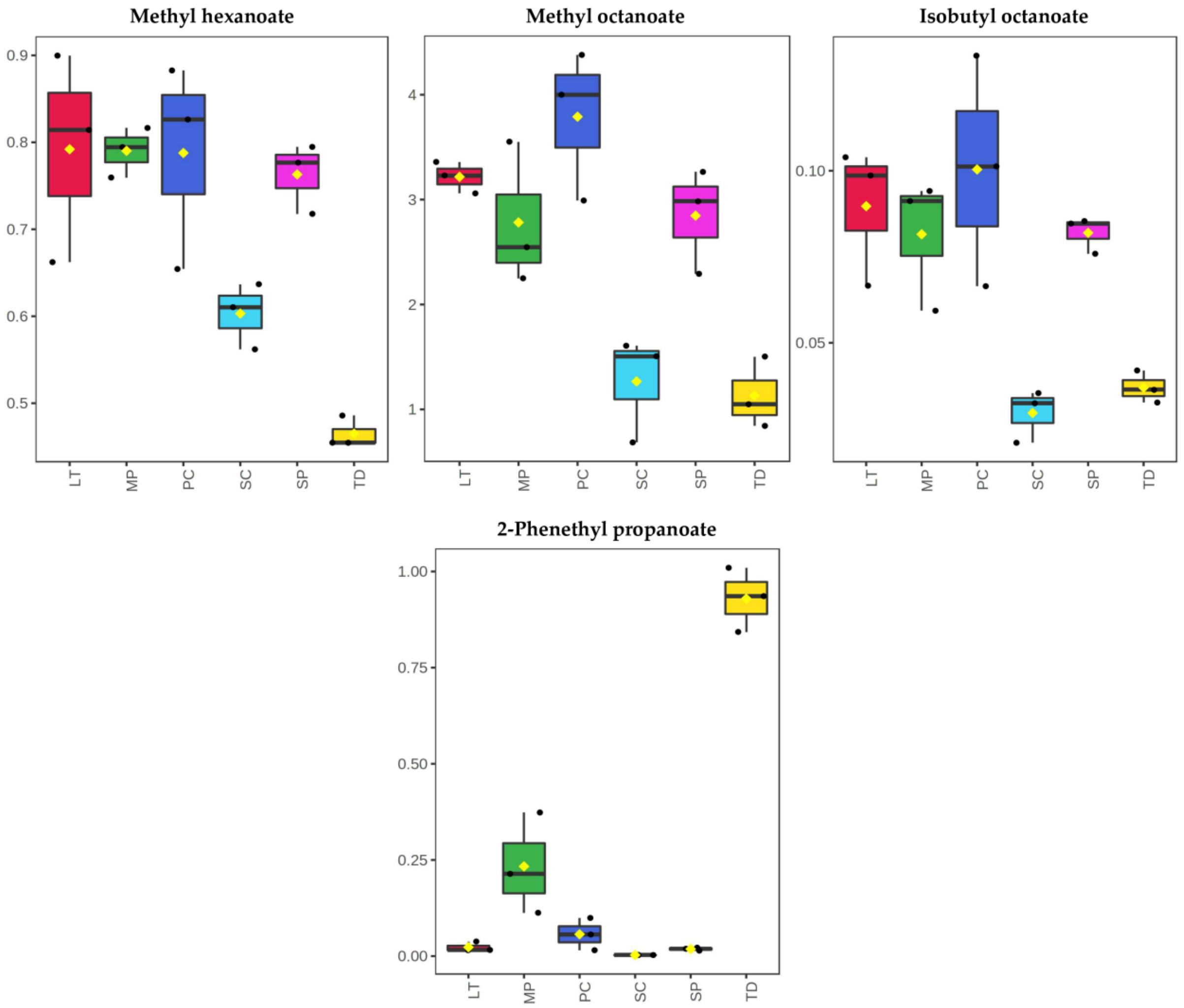

Figure S5. Concentrations of other esters with high differentiating ability among yeasts (high *F*-ratios) identified in the early phase of fermentation of Malvazija istarska grape must inoculated by *Saccharomyces cerevisiae* and five non-*Saccharomyces* yeasts. Abbreviations: SC – *Saccharomyces cerevisiae*; TD – *Torulaspora delbrueckii*; MP – *Metschnikowia pulcherrima*; PC – *Pichia kluyveri*; LT – *Lachancea thermotolerans*; SP – *Schizosaccharomyces pombe*.

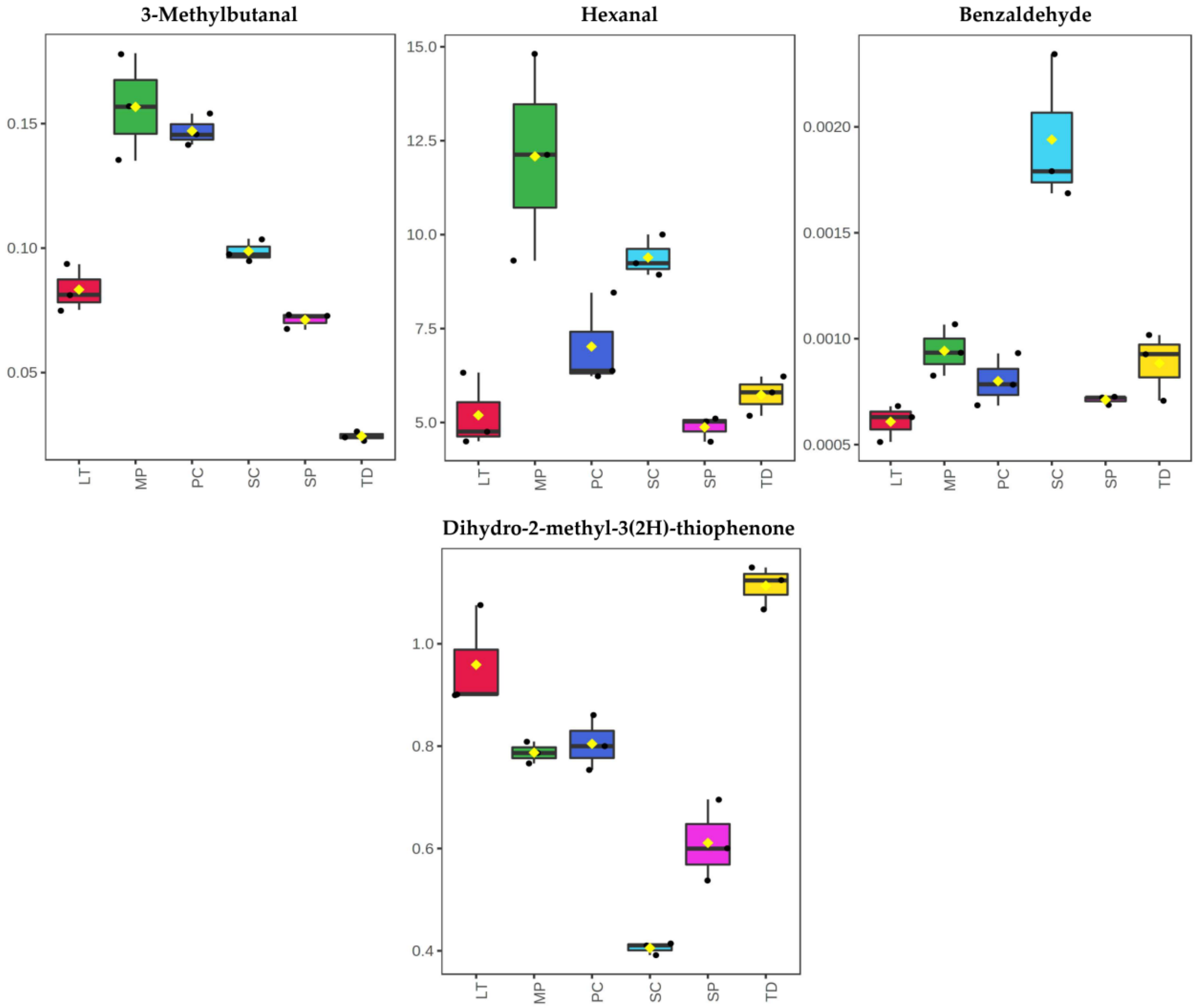

Figure S6. Concentrations of miscellaneous volatile compounds with high differentiating ability among yeasts (high *F*-ratios) identified in the early phase of fermentation of Malvazija istarska grape must inoculated by *Saccharomyces cerevisiae* and five non-*Saccharomyces* yeasts. Abbreviations: SC – *Saccharomyces cerevisiae*; TD – *Torulaspora delbrueckii*; MP – *Metschnikowia pulcherrima*; PC – *Pichia kluyveri*; LT – *Lachancea thermotolerans*; SP – *Schizosaccharomyces pombe*.
